# Differential Gene Expression and Metabolic Pathway Analysis of *Cladophora rupestris* under Pb Stress Conditions

**DOI:** 10.3390/ijerph192113910

**Published:** 2022-10-26

**Authors:** Lei Liu, Lusheng Zhang, Lingyun Zhao, Qiuyu Chen, Qian Zhang, Deju Cao, Zhaowen Liu

**Affiliations:** 1 Anhui Province Key Laboratory of Farmland Ecological Conservation and Pollution Prevention, School of Resources and Environment, Anhui Agricultural University, Hefei 230036, China; 2 School of Materials and Environmental Engineering, Chizhou University, Chizhou 247000, China

**Keywords:** *C. rupestris*, Pb, transcriptome, DEGs, transporter proteins, MT, GSH

## Abstract

This study aimed to analyze the transcriptome of *C. rupestris* under Pb^2+^ stress by using high-throughput sequencing technology, observe the changes of gene expression and metabolic pathway after three and five days under 1.0 and 5.0 mg/L of Pb^2+^ treatment, and analyze the differentially expressed genes (DEGs) and related functional genes after Pb^2+^ treatment. Metabolic pathways were revealed through Gene Ontology (GO) and the Kyoto Encyclopedia of Genes and Genomes (KEGG) pathway analysis. Results show that DEGs increased significantly with the increase of Pb^2+^ concentration and stress time. A total of 32 genes were closely related to Pb^2+^ stress response. GO analysis identified two major transporter proteins, namely, ATP-binding transport protein-related (ABC transporters) and zinc finger CCHC domain containing protein (Zfp) in *C. rupestris*. Pthr19248, pthr19211, Zfp pthr23002, Zfp p48znf pthr12681, Zfp 294 pthr12389, and Zfp pthr23067 played important roles against Pb^2+^ toxicity and its absorption in *C. rupestris*. KEGG pathway analysis suggested that ABCA1, ATM, and ABCD3 were closely related to Pb^2+^ absorption. Pb^2+^ stress was mainly involved in metallothionein (MT), plant hormone signal transduction, ABC transporters, and glutathione (GSH) metabolism.

## 1. Introduction

Lead (Pb) is one of the most toxic pollutants and is a persistent environmental contaminant [1]. Some plants such as *Rhus chinensis* Mill (*Anacardiaceae*) and *Conyza canadensis* could overaccumulate heavy metals (HMs) [2,3]. *Cladophora rupestris* has a strong ability to absorb and tolerate Pb^2+^ stress and alter the distribution of Pb at both the tissue and sub-cellular levels to reduce toxicity [4,5]. However, the tolerance and detoxification mechanisms of hyperaccumulate were complex [6,7,8,9]. Pb^2+^ enter into the *Chladophora* cells by forming chelate with GSH/MT and –OH functional groups [7], and HMs transport plays an important role in the detoxification of plants by sequestering toxic or excess HMs into the vacuoles [10]. The defense mechanism of *Cassia alata* is based on Cd accumulation in roots, coupled with an increase in GSH [11]. SpHMA1 functions as a function of chloroplast Cd exporter and prevents Cd accumulation; the hyperexpression of SpHMA1 contributes to Cd hypertolerance in *S. plumbizincicola* [12]. MT could enhance Pb accumulation in *Cladophora* [6]. The expression of MT, ATP-binding cassette (ABC) transporter, and zinc and manganese transporter genes depend on both the concentration of cadmium and exposure time [13]. ABC transporters and HMs ATPases (HMA) are involved in Cd transportation [14].

High-throughput RNA sequencing (RNA-seq) is rapidly emerging as a major quantitative transcriptome profiling platform, identified as salt-responsive genes by using transcriptome sequencing in *Dunaliella viridis* [15]. Li, Y. et al. sequenced and analyzed the transcriptome of Tree Penoy Petals by using RNA-seq and investigated the molecular response of tree peony petals for grafting with two different rootstocks [3]; comparative transcriptome analysis was used to provide new insights into the mechanism of biosorption of Cu^2+^ [16]. Transcriptomic and physiological analyses were conducted to help understand the mechanism of cadmium accumulation and detoxification in *P. canescens* [17].

*C. rupestris* has some adsorptive ability and tolerance to Pb^2+^ [5]. Based on our previous research, *Cladophora* could accumulate Pb^2+^ but not Zn^2+^ and Cu^2+^ [4,6]. The mechanism underlying Pb^2+^ detoxification by *C. rupestris* is interesting, but the mechanism remains unclear.

In the present study, we analyzed the transcriptome of *C. rupestris* under Pb stress by using high-throughput sequencing technology. Comparative transcriptome analysis of cells was conducted under different concentrations of Pb treatment and stress time. DEGs were analyzed, and the related functional genes and metabolic pathways were revealed through GO and KEGG analysis. These processes were conducted to clarify the difference in transcriptional responses to Pb. Furthermore, understanding of the genetic basis underlying these physiological processes would help reveal the mechanism of Pb biosorption and tolerance.

## 2. Materials and Methods

### 2.1. C. rupestris Cultivation and Preparation

*C. rupestris* was collected from surface water of a pond in Hefei in the Anhui Province, China (31°50′ N, 117°11′ E). The configuration of the culture medium of *C. rupestris* was Bold’s Basal Medium (BBM), and the samples were grown in a sterile incubator at 25 °C (SPX-250B-G) with light intensity ranging from 3000 to 4000 lx (light and dark cycle of 12:12 h) and cultured at a pH value of approximately 7.5 ± 0.5 [4,6].

### 2.2. Experimental Design

Then, 2.0 g samples were grown in 1000 mL BBM and contained various concentrations of Pb(NO_3_)_2_. Pb concentrations were set as 0, 1, and 5.0 mg/L, while the Pb stress time was set as 3 and 5 days [7,8,9]. The samples were numbered as Pb1_3 and Pb1_5 for the 1.0 mg/L treatment group on the third and fifth day, and Pb5_3 and Pb5_5 for the 5 mg/L treatment group on the third and fifth day, respectively. Three biological replicates were set up for each group.

### 2.3. Total RNA Extraction

After 3 and 5 days, *C. rupestris* samples were collected and washed with deionized water. Liquid nitrogen was added to 80 mg of *C. rupestris* cells and ground into powder, total RNA was isolated from *C. rupestris*, Ethanol precipitation protocol and CTAB-PBIOZOL reagent were used for the purification of total RNA according to the manual instructions [18].

### 2.4. mRNA Library Construction

mRNA was purified from total RNA by using Oligo(dT)-attached magnetic beads. Purified mRNA was fragmented into small pieces with fragment buffer (0.5 mol/L NaCl, 20 m mol/L Tris-HCl (pH 7.6) at 94 °C. First- and second-strand cDNA synthesis was generated using Shah’s method [19].

Microarray analysis was carried out using the Agilent Technologies 2100 Bioanalyzer. Microarray hybridization, washing, staining, scanning, and data processing were conducted by Beijing Genomics Institute (BGI, BGI-Shenzhen, China).

### 2.5. Differential Expression Analysis

The original sequencing data contained reads with low quality, joint contamination, and high unknown base nitrogen content. These reads were removed before data analysis to ensure the reliability of the results. For samples lacking reference genomes, clean reads were assembled in reference to Shah’s method to obtain a reference sequence [19]. Tgicl was used to cluster the transcript to remove redundancy. The DEG screening method is based on Poisson distribution, and all the data from Pb^2+^ treatments were quantified as fold change (FC) compared with the control [20]. Genes were considered screened as DEGs when log2 FC ≥ 2 and QP-value ≤ 0.001.

### 2.6. Data Analysis

The DEGs were classified and grouped by GO functional (http://geneontology.org. accessed on 1 January 2021) and KEGG pathway (http://www.genome.jp/kegg, accessed on 1 January 2021). Then, FDR correction was carried out for *p* value, and significant enrichment was considered at *Q* value ≤ 0.05.

The data were analyzed using Excel 2019 to average the sum of three parallel samples and expressed as mean ± standard deviation (SD). The DEGs were statistically analyzed with one-way ANOVA (*p* < 0.05). The Duncan test was conducted with SPSS18.0, and were tested using Pearson’s coefficient to determine the presence of positive or negative correlations at * *p* < 0.05 or ** *p* < 0.01. Originpro8.5 software was used to analyze data and create diagrams.

## 3. Results

### 3.1. Quality Evaluation of Sequencing Reads

The quality assessment of sequencing reads was evaluated before the subsequent bioinformatics analysis. As shown in Table 1, the filtered reads for each group were approximately 43 M, and the percentage of clean reads for each group was over 93%. Q20 and Q30 refer to the proportion of base with Phred scores >20 or 30 in the total bases, and these values were calculated to determine the quality of clean data [18,21]. A high score ensures the accuracy and data quality. The average readings of the filtered Q20 and Q30 were approximately 97% and 90% of the original readings, respectively (Table 1). These findings indicate low quality base ratios and good sequencing quality for further Denovo assembly [21]. Trinity assembly results show that each group had an N50 of over 1200, and Guanine and cytosine (GC) content of approximately 46%; no significant difference was observed between the control and treatment groups, indicating high assembly quality and can be used for subsequent analysis [13,20].

### 3.2. Identification of DEGs Responding to Pb^2+^

#### 3.2.1. Number of DEGs

**Figure 1:**
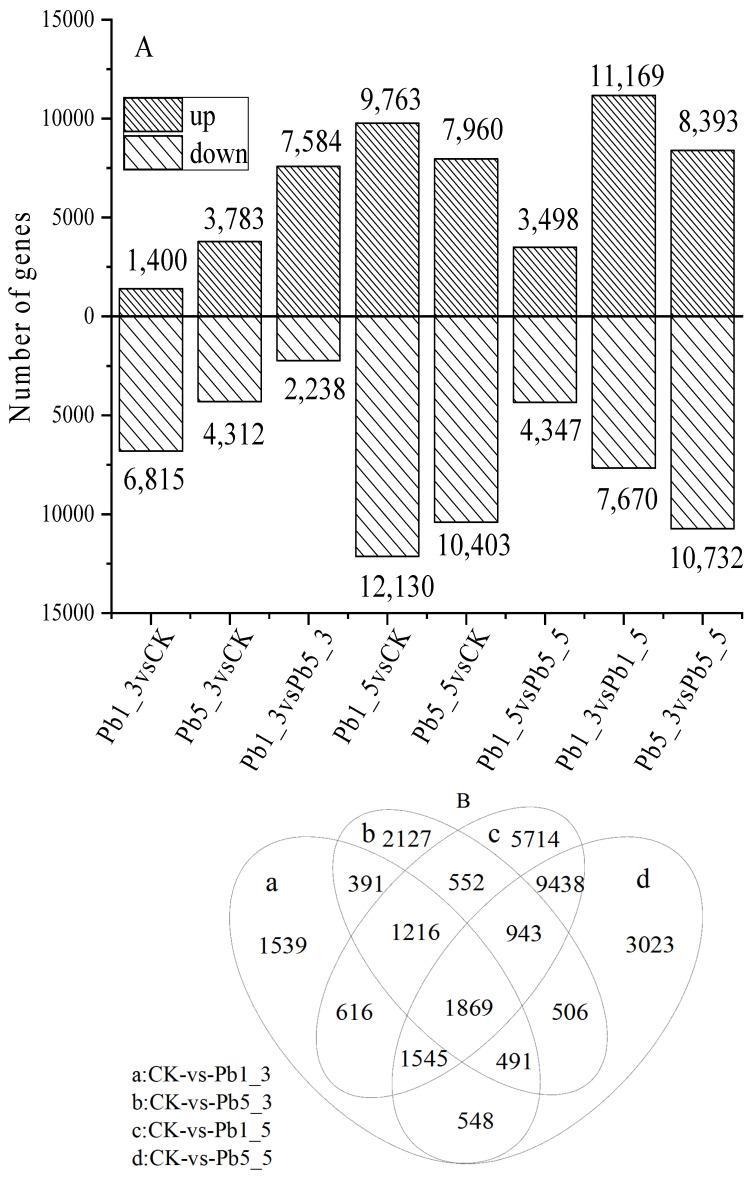
DEGs of *C. rupestris* under Pb^2+^ stress. Note: (**A**) under a different concentration of Pb; (**B**) Venn diagram (Common DEGs).

DEGs with a difference multiple of more than two and a Q value ≤ 0.001 were selected as significantly differential expressed genes [20]. As shown in Figure 1A, differential analysis showed that 21,893 genes were differentially expressed in *C. rupestris* under Pb^2+^ treatment.

According to Figure 1A, DEG analysis showed that compared with the control group, the expression levels of 1400 unigenes in *C. rupestris* exposed to 1.0 mg/L of Pb^2+^ at day 3 were upregulated, whereas the expression levels of 6815 unigenes were downregulated, and while the expression levels of 3783 were upregulated and 4312 were downregulated when exposed to 5.0 mg/L of Pb for 3 days, this time *Cladophora* could accumulate Pb [6], and the Pb concentration in the cell wall was 558.9 and 1661.2 mg/kg, the organelle was 231.5 and 286.3 mg/kg, the soluble fraction was 103.2 and 258.6 mg/kg when the Pb level was 1.0 mg/L and 5.0 mg/L [20]. The number of upregulated genes increased significantly with the increase of Pb^2+^ concentration, reaching 3783, and these genes may play important roles in tolerance to Pb stress [18]. Therefore, Pb stress at a 5.0 mg/L concentration had a more obvious effect than that at a 1.0 mg/L concentration on *C. rupestris* [18]. These DEGs may play a key role in the stress process [13,15]. The comparison results imply the differences in gene expression in *C. rupestris* exposed to Pb^2+^, and the number of DEGs increased significantly with the increase of Pb^2+^ concentration, and this result was consistent with the findings of Cao et al. and Chen et al. [6,8,9].

Furthermore, Pb^2+^ exposure time had a considerabe effect (Figure 1A). A total of 11,169 upregulated and 7670 downregulated genes were differentially expressed in 1.0 mg/L Pb^2+^ after 3 and 5 days, and 8393 upregulated and 10,732 downregulated genes were differentially expressed in 5.0 mg/L Pb comparisons 3d and 5d. DEGs increased significantly with the increase of Pb^2+^ stress time, indicating a significant difference in stress time of Pb to *C. rupestris*, and 1869 common DEGs were found in the Pb1_3vsCK, Pb1_5vsCK, Pb5_3vsCK and Pb5_5vsCK groups (Figure 1B); these DEGs play a key role in the stress process [13].

#### 3.2.2. Functional Annotation of MT Related Genes

As shown in Table 2, DEGs were annotated through Swissprot (https://www.uniprot.org/, accessed on 15 March 2021) and Pfam (http://pfam.xfam.org, accessed on 15 March 2021) databases, and eight MT-related genes were annotated. Three MT genes were upregulated, including the PEC family and two MTs, indicating that CL7373.Contig1_All (FC was 1.64), Unigene37386_All (FC was 3.5), and Unigene2683_All (FC was 1.39) might play a key role in the *C. rupestris* response to Pb stress, and these genes can be used as key candidate genes [13].

#### 3.2.3. GO Enrichment Analysis of Functional DEGs

**Figure 2:**
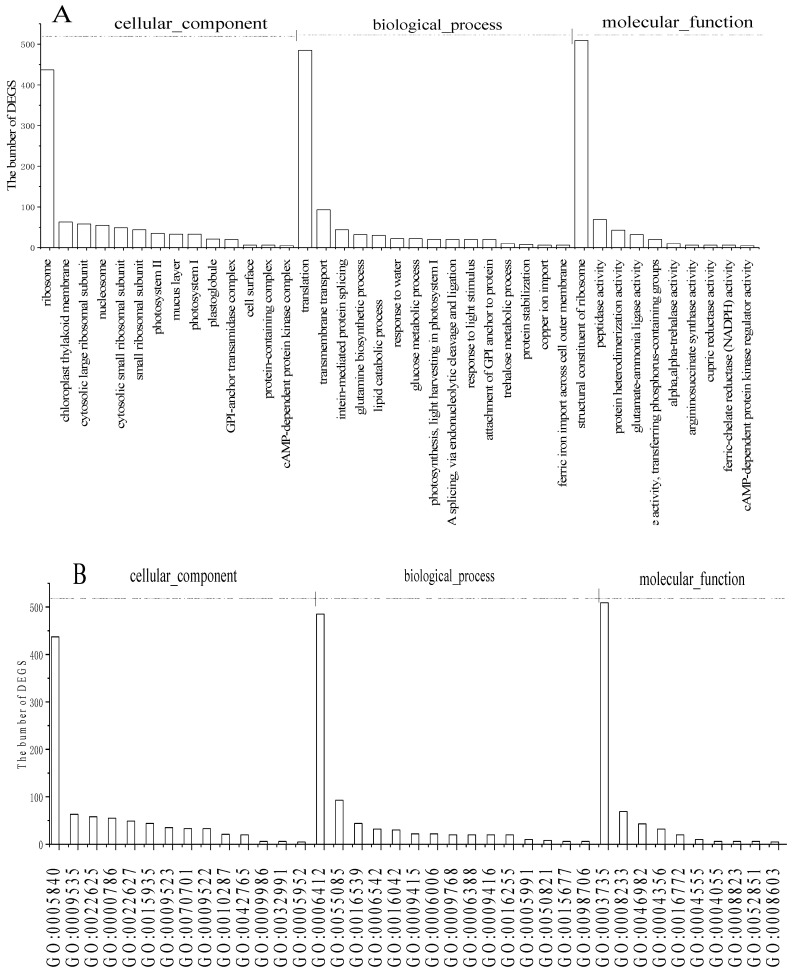
GO annotation of the DEGs. (**A**) GO classification of the DEGs. (**B**) GO term ID of the DEGs).

The GO enrichment analysis results of DEGs in Pb1_5 vs. Pb5_5 were classified according to molecular functions (MF), biological processes (BP), and cell components (CC). To identify the candidate genes for Pb^2+^ stress resistance in *C. rupestris*, the GO items with the most significant (filtering with *Q* value ≤ 0.05) enrichment were selected from each GO classification for analysis (Figure 2A).

The terms of ribosome (GO:0005840) were dominant in cell components; those of structural constituent of ribosome (GO:0003735) and peptidase activity (GO:0008233) were mainly involved in molecular function, while those of translation (GO:0006412) and transmembrane transport (GO:0055085) were mainly involved in biological processes.

As shown in Figure 2, the ribosome and structural constituents of ribosome were dominant in CC and MF, respectively, and this indicated that the gene about protein synthesis and regulation was significantly affected by Pb^2+^ stress [22]. In addition, peptidase activity, protein-containing complex, cAMP-dependent protein kinase complex, intein-mediated protein splicing, glutamine biosynthetic process, protein stabilization, protein heterodimerization activity, glutamate-ammonia ligase activity, and cAMP-dependent protein kinase regulator activity and the like polypeptide-associated genes were significantly affected by Pb^2+^ stress. MT concentration in *Cladophora* increased as the Pb concentration increased [6]. Non-protein thiols (NPT), GSH, and phytochelatins (PCs) increased significantly with increasing Pb stress in the cell wall and soluble fraction [8], suggesting that MT, NPT, GSH, and PCs are important candidate genes for Pb^2+^ stress resistance in *C. rupestris* [6,8]. Genes related to GSH (GSH1 and GSH2) and PCs (PCS1 and PCS2) were also increased in apx1-3 plants subjected to Pb stress [23].

Genes related to photosynthesis, such as chloroplast thylakoid membrane (GO:0009535), photosystem I (GO:0009522), photosystem Ⅱ (GO:0009523) in CC, photosynthesis, light harvesting in photosystem I (GO:0009768), and response to light stimulus (GO:0009416) in BP (Figure 2), were significantly affected by Pb^2+^ stress, indicating that the photosynthesis of *C. rupestris* was influenced by Pb^2+^ stress, and the controlling genes were GO:0009535, GO:0009522, GO:0009523, GO:0009768, and GO:0009416. Cao et al. reported that photosynthesis is highly sensitive to Pb, and chlorophyll content is negatively correlated with Pb^2+^ concentration, supporting the experimental results in this paper [6].

Transfer-related genes, such as transmembrane transport, structural constituent of ribosome, copper ion import, ferric iron import across cell outer membrane, transferase activity, and transferring phosphorus-containing groups were significantly affected by Pb^2+^ stress (Figure 2). We identified two ABC transporter genes, including ATP-binding transport protein-related pthr19248 (EUGRSUZ_A00112; EUGRSUZ_A00046;A0A2I2YPV7; ABC transporter E family member 2; Kcr_0070; 103639168; 101778969; UMAG_03351; LOC100184673; BRADI_3g33470v3; LOC104606445; 100217065; GSPATT00016840001; EUGRSUZ_A00294; CL6EHI_156200; CL6EHI_150540; EHI_150530; W5MR47; ATRLI2; TVAG_249850; A0A287NTT5; A0A287JRE8; Q93YY0; M1A0G7; THAPSDRAFT_bd1652; POPTR_004G235900; 100804088; SELMODRAFT_413898; PRUPE_1G366500;PRUPE_3G017900; SORBI_3004G128100; SS1G_06617;AMTR_s00066p00025940 and so on), and ATP-binding transport protein-related pthr19211(UMAG_04152;CND02420 and the like), which was the largest subfamily and the dominant ABC transporter gene subfamily in *C. rupestris*. The ABC-B1 subfamily group proteins from systems involved in the uptake of organic and inorganic ions enhanced Pb tolerance mainly by activating the expression of the ABC-type transporters [23,24]. Most flax ABC transporters participate in ATP binding, transport, ATPase activity, and metal ion binding [25]. These genes are the most likely candidates for Pb accumulation in *C. rupestris*.

Transcription factors of Zfp are differentially expressed during Pb^2+^ stress to *C. rupestris*, and four kinds of transcription factors might be associated with Pb.Zfp pthr23002(A0A2I2YNE6;UMAG_11314; v1g120512; ZCCHC13; SNOG_11691; UMAG_11258; BRADI_4g18203v3; POPTR_010G092600; SORBI_3009G088550; SORBI_3009G098801; SORBI_3009G080150; SORBI_3009G085250; SORBI_3009G133201; SORBI_3009G072366; SORBI_3009G099450; SORBI_3009G109775; SORBI_3009G092850; SORBI_3009G085051; SORBI_3009G071401; SORBI_3009G080266; SORBI_3009G064250; CNBP; NCU05800; LOC107769619; W5N9C9; ZCCHC13; ANIA_05111; LOC107796219; Tb11.01.1270; PRUPE_1G269000; PGTG_10593;SORBI_3001G275950; 100823164; SORBI_3001G384900; H3GI81; LSAT_4X8860; CL6EHI_055640; CNE02090; SORBI_3009G091980; SORBI_3009G095480; SORBI_3009G095560; DAPPUDRAFT_112160; A0A2I2YYV6; 101784076; CNBP; ZEAMMB73_Zm00001d011718; GSPATT00016774001; GLYMA_18G125100; SELMODRAFT_450823; F7EJV2; SS1G_04861 and so on), Zfp p48znf pthr12681(F2E763; EUGRSUZ_A01194; YALI0_D07018g; LOC107791523; ANIA_06922; HannXRQ_Chr17g0559121; LSAT_8X20961; CNK01650; W4Z699; ZC3H15; GSPATT00004747001; POPTR_005G167200; 100783113; F6W619; SORBI_3001G353600; SORBI_3003G346700; THAPSDRAFT_4543; BRAFLDRAFT_288490; PF3D7_1244800; PRUPE_1G511400 and so on), Zfp 294 pthr12389(G3QDL6; ltn1; W5MRG2; CL6EHI_190430; CNE03250; LOC104597233;G3QDL6; 8030755; ANIA_06404; TVAG_343770; TCM_042247; A0A3B6N2V3; AMTR_s00007p00201600; 29273;BATDEDRAFT_33847; 100775576; SS1G_07566 and so on), and Zfp pthr23067(A0A2I2ZRX7). An HM transporter cDNA, ZNT1(Zfp), was cloned from *Thlaspi caerulescens* through functional complementation in yeast and mediated high-affinity Zn and low-affinity Cd uptake [26]. Our results were consistent with previous reports on annotated sequences matching with Zfp proteins of *C. rupestris*, indicating that the protein regulated the absorption and migration of Pb^2+^ in *C. rupestris* [26]. The genes may play important roles in the Pb absorption of *C. rupestris* [6].

### 3.3. KEGG Analysis of DEGs in C. rupestris under Pb Stress

**Figure 3:**
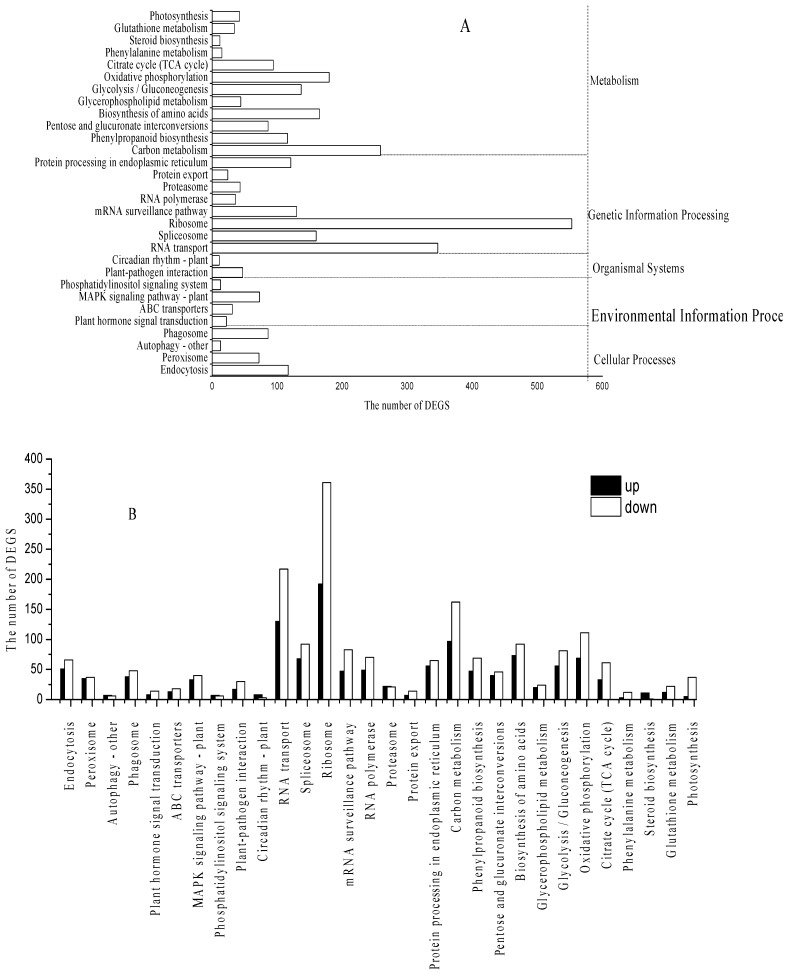
KEGG analysis of the identified DEGs. (**A**) was KEGG classification of DEGs; (**B**) was upregulated and downregulated DEGs of every pathway.

KEGG analysis was performed to characterize the pathway enrichment of the identified DEGs, and these genes were classified into including cellular processes, organismal systems, environmental information processing, genetic information processing, and metabolism. A total of 7845 differential genes were observed in Pb5_5vsPb1_5, and 2919 differential genes were annotated to 121 KEGG pathways, among which 30 metabolic pathways were significantly enriched (Figure 3A).

#### 3.3.1. DEGs in ABC Transporters

A total of 31 DEGs of ABC transporters (ko02010) were detected from *C. rupestris* treated with 1.0 and 5.0 mg/L of Pb. Thirteen genes were upregulated, and 18 genes were downregulated (Figure 3B), including two ABCA1 genes, one ABCA3 gene, two ABCB1genes, one ABCB8 gene, 15 ABCC1 genes, two ABCC2 genes, one ABCD3 gene, six ABCG2 genes, and one ATM (mitochondrial ABC transporter) gene. Among them, 17 DEGs include CL2046. Contig1_All, CL2046.Contig2_All, Unigene15678_All, and Unigene28062_All were upregulated (|log2FC| ≥ 2, Table 3). These genes were associated with absorption of Pb in *C. rupestris* [27].

ABC transporter proteins were present in almost all prokaryotic and eukaryotic cells, and many ABC genes were involved in HM tolerance [24,28]. In the present study, the induction of ABCC1 and ABCG2 transporter proteins was more pronounced during Pb^2+^ stress in *C. rupestris*, and ABCA1, ATM, and ABCD3 were upregulated when the Pb^2+^ concentration was increased (Figure 3A), while ABCC2, ABCB1, ABCB8, and ABCB10 were all downregulated. Besides, ABCA3, ABCC1, and ABCG2 were up- and down-regulated. All 31 ABC transporter proteins belong to six subfamilies, namely, ABCA, ABCB, ABCC, ABCD, ABCG, and ATM; ABCB, ABCC, and ABCG played important roles in HM detoxification [29]. The differential expression of ABC transporters in *C. rupestris* under Pb^2+^ stress suggests that it needs to regulate its tolerance through up- or downregulation of transporter protein activity [28]. Hence, ABCC1 and ABCG2 transporter proteins were more pronounced during Pb^2+^ stress in *C. rupestris*, and ABCA1, ATM, and ABCD3 were also closely related to Pb^2+^ absorption.

#### 3.3.2. Identification of Genes in GSH Metabolism

Based on KEGG pathway analysis (Figure 3B), 34 DEGs were identified and were involved in several processes in the GSH metabolism, and 17 DEGs had log2Fold change ≥ 2. These genes encoded different types of enzymes, such as isocitrate dehydrogenase, glucose-6-phosphate 1-dehydrogenase, 6-phosphogluconate dehydrogenase, glutathione S-transferase, and ribonucleoside-diphosphate reductase subunit M2. CL168.Contig2_All, CL3367.Contig4_All, Unigene17078_All, Unigene28066_All, Unigene53785_All, Unigene54130_All, Unigene55336_All, and Unigene55337_All were upregulated. These genes were associated with plant defense response to Pb stress in *C. rupestris* (Table 3).

GSH plays a central role in the plant tolerance against metals, can bind and help Pb^2+^ enter the cell, and was a key antioxidant that acts as a cofactor for glutathione S-transferase [9,30]. GSH was the most important non-enzymatic intracellular defense mechanism against reactive oxygen species (ROS) and controls the plant response to toxic elements [9]. In addition, it acts as a substrate for divalent cations such as Pb^2+^, where GSH acts as a precursor for PCs and as a substrate for GSH metabolizing enzymes to protect the -SH group in the enzyme [31]. In the present study, 34 DEGs were annotated into the GSH metabolic pathway and were mainly divided into dehydrogenase and GSH S-transferase. The formation of GSH-Pb conjugate (GS-Pb) is dependent on GSTs and plays an important role in Pb^2+^ detoxification [9,32]. The upregulation of GSH transferase helped in promoting the formation of GSH-Pb, which enhances Pb^2+^ detoxification (GS-Pb). In addition, ABCC proteins transport substrates act as GSH conjugates [33]. Therefore, upregulated changes in ABCC transporter proteins contribute to the transport of GSH conjugates. In summary, Pb binding to GSH was catalyzed by glutathione transferase, followed by GS-Pb binding to the vesicle via the ABCC transporter [32].

#### 3.3.3. Identification of Peroxidase-Related Genes

As shown in Figure 3B, 72 DEGs were annotated into the peroxisomal pathway through KEGG enrichment analysis, 35 genes were upregulated, 37 genes were downregulated, and 10 of these genes are involved in the encoding of superoxide dismutase (SOD), catalase (CAT), epoxide hydrolase, and a class of peroxidases, which may play a key role in oxidative stress of *C. rupestris* under Pb stress [6].

Under Pb^2+^ stress, SOD and peroxidase (POD) enzyme activities in *Pogonatherum crinitum* leaves were increased, which could reduce the cell membrane damage [34]. SOD and CAT in *Datura stramonium Linn* under Cd stress showed an increasing trend at the beginning of the treatment and gradually decreased with time. Excessive Cu stress increased SOD and ROS activity in *Closterium ehrenbergii* cells [28]. In the present study, 72 DEGs were annotated to the peroxisomal pathway, and 10 DEGs were identified as related to SOD, CAT, and other enzymes, which may be related to the oxidative stress response in the *C. rupestris* response to Pb stress [6].

#### 3.3.4. Identification of Phenylpropanol Biosynthesis Gene

A total of 116 DEGs were annotated into a phenylpropanol synthesis pathway (Figure 3B), and 47 DEGs were upregulated and 69 were downregulated. Phenylpropanol is a natural plant compound commonly extracted from phenylalanine [35], which plays an important role in the response of plants to a variety of biotic and abiotic stresses [36]. Cinnamyl alcohol dehydrogenase and cinnamoyl coenzyme reductase were identified from DEGs (Table 2). Cinnamyl alcohol dehydrogenase included Unigene55182_All, Unigene54923_All, and Unigene53862_All; and cinnamoyl coenzyme a reductase included Unigene53942_All, Unigene13418_All, and Unigene10106_All. In addition, the branching biochemical reactions of phenylpropanoid biosynthesis, and protect plants from Pb-induced oxidative stress by scavenging H_2_O_2_ and reactive free radicals [37].

#### 3.3.5. Hormone Signaling and Mitogen-Activated Protein Kinase (MAPK) Signaling Genes

As shown in Table 3, two genes were identified, which were related to growth hormone among plant hormones (Unigene34841_All and CL263.Contig32_All), while many genes were involved in MPAK transduction, and two representative genes (Unigene3664_All and Unigene55189_All) were selected with FC ≥ 2. Environmental stressors are delivered to target transcription factors via the hormone signaling pathway and MAPK signaling pathway [38]. HM-related hormones include abscisic acid, growth hormone, jasmonic acid, and salicylic acid [39]. Pb-organic acids resist the toxicity of Pb^2+^ [9]. The expression of MAPKs and GSH metabolism genes was upregulated under HM stress [40]. The MAPKs signaling pathway plays a key role in regulating crosstalk between plant signaling systems as a means of facilitating adaptation, and coordination of plant responses to various stressors [41].

### 3.4. Pb Absorb and Response Pathways in C. rupestris

**Figure 4:**
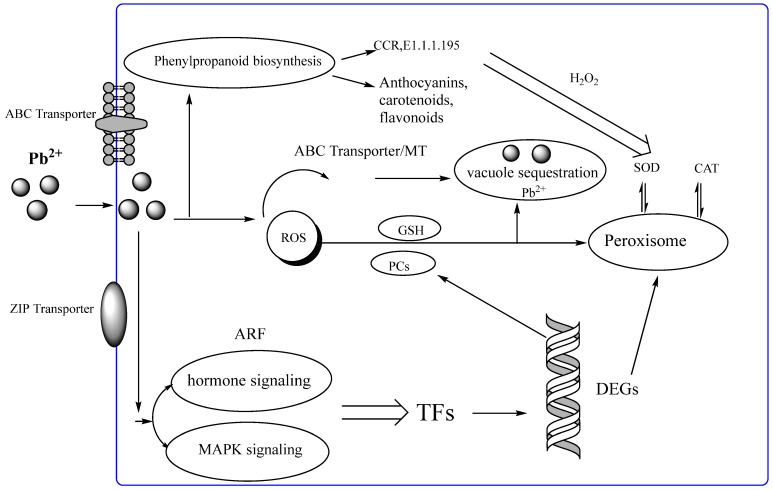
Pb absorb and emergency response of *C. rupestris.* Note: CCR: cinnamoyl-CoA reductase; E1.1.1.195:cinnamyl-alcohol dehydrogenase; GSH, glutathione; TF, transcription factors; ROS, reactive oxygen species; ARF; auxin response factor; DEGs, differentially expressed genes; MT: Metallothionein; PCs: phytochelatins.

As shown in Figure 4, combined with the above analysis, when Pb^2+^ entered the cell via a transporter such as ABC transporters and zinc finger protein [15], the metal transporter genes, AtACBP1and AtHMA3, in Arabidopsis are associated with the transportation of Pb [42]; the FLS1 gene plays a crucial role in plant resistance to Pb stress [43].

Pb^2+^ was sent to the vesicles’ storage and isolation by MT, GSH, PCs, and Pb-organic acids to resist the toxicity of Pb^2+^ in *C. rupestris*, and SOD and CAT were increased through the expression of peroxidase genes. MePMEI1 gene increases heavy metal tolerance [44]. On the other hand, Pb^2+^ affects the phenylpropanoid biosynthesis and hormone cellular transmission processes in *C. rupestris*, which involve the synthesis of anthocyanins, carotenoids and flavonoids, Pb-induced oxidative stress by scavenging H_2_O_2_ and reactive free radicals [37]. At the same time, Pb^2+^ entering a cell causes changes in phytohormone concentrations, induced of GSH, via hormone signaling, MAPK signaling, and ROS interacts with phytohormones, protection against and mitigation of Pb stress [20].

## 4. Conclusions

The filtered reads for each group were approximately 43 M, and the percentage of clean reads for each group was over 93%. Q20and Q30 were approximately 97% and 90% of the original readings respectively; this indicates high assembly quality and can be used for subsequent analysis.

Pb stress caused changes in DEGs that increased significantly with the increase of Pb^2+^ concentration and stress time. Moreover, 35 genes closely related to Pb stress response were screened. Three metallothionein-related genes were identified as candidate genes in the Swissprot and Pfam databases, namely, CL7373.Contig1_All, Unigene37386_All, and Unigene2683_All. GO analysis identified two major transporter proteins, namely, ABC transporters and Zfp in *C. rupestris*. Pthr19248, pthr19211, Zfp pthr23002, Zfp p48znf pthr12681, Zfp 294 pthr12389, and Zfp pthr23067 played important roles against Pb infection and its absorption in *C. rupestris*.

KEGG pathway analysis showed that ABC transporter proteins belong to six subfamilies, namely, ABCA, ABCB, ABCC, ABCD, ABCG, and ATM, with some members of the subfamilies ABCB, ABCC, and ABCG playing important roles in Pb detoxification in different species. Moreover, ABCA1, ATM, and ABCD3 were closely related to lead absorption. Pb binding to GSH was catalyzed by glutathione transferase, followed by GS-Pb binding to the vesicle via the ABCC transporter. In addition, the analysis of the KEGG metabolic pathway indicates that glutathione metabolism, phenylpropanoid synthesis, hormone signaling and MAPK signaling pathways are important in the defense mechanism of *C. rupestris* under lead stress. A total of 32 key genes were also screened. This research will contribute to the development and study of HM remediation techniques for aquatic plants in the future.

## Figures and Tables

**Table 1:** The reads and the quality index of Unigenes.

Sample	Filtered Reads (Mb)	Q20(%)	Q30(%)	Filtered Reads Radio (%)	N50	GC (%)
CK	42.5 ± 0.2 a	97.3 ± 0.1 a	90.1 ± 0.3 a	93.4 ± 0.440 a	1534 ± 75 ab	46.3 ± 0.4 a
Pb1_3	42.5 ± 0.2 a	97.2 ± 0.1 a	89.7 ± 0.3 a	93.341 ± 0.4 a	1584 ± 28 b	46.1 ± 0.5 a
Pb1_5	43.2 ± 0.1 b	97.2 ± 0.2 a	89.8 ± 0.5 a	94.7 ± 0.3 b	1346 ± 156 a	46.7 ± 0.9 a
Pb5_3	43.3 ± 0.1 b	97.2 ± 0.0 a	89.8 ± 0.1 a	95.0 ± 0.3 b	1533 ± 132 ab	46.0 ± 0.700 a
Pb5_5	43.3 ± 0.2 b	97.4 ± 0.4 a	90.3 ± 0.9 a	95.1 ± 0.4 b	1380 ± 131 ab	46.101 ± 0.3 a

Note: ”±” indicates mean ± standard deviation, and different letters in each column indicate differences between treatment groups (same letters indicate non-significant differences *p* > 0.05, different letters indicate significant differences *p* < 0.05, Duncan); Q30, Q20 refers to the proportion of base with Phred scores >30 or 20 in the total bases. A high score ensures the accuracy and data quality.

**Table 2:** Metallothionein-related genes.

Gene	Fold Change	Subject Annotation
CL7373.Contig1_All	1.64	Metallothionein
Unigene11338_All	−1.2	Yeast metallothionein
Unigene23676_All	---	Metallothionein
Unigene2683_All	1.39	Plant PEC family metallothionein
Unigene37386_All	3.5	Metallothionein
Unigene8627_All	−1.0	Metallothionein
Unigene36067_All	---	Metallothionein-like protein
Unigene47760_All	---	Metallothionein-like protein

Note: The differential multiple is positive, which means that the expression level is upregulated; the differential multiple is negative, which means that the expression level is downregulated; the differential multiple is ---, which means that there is no change before and after.

**Table 3:** Candidate gene of KEGG analysis.

Pathway Name	Pathway Id	Up Genes	Note
ABC transporters	ko02010	CL189.Contig3_All (ABCA1) CL189.Contig9_All (ABCA1) Unigene3596_All (ABC.ATM) Unigene42923_All (ABCG2)	ABCA1: ATP-binding cassette, subfamily A (ABC1), member 1 ABC.ATM: mitochondrial ABC transporter ATM ABCG2: ATP-binding cassette, subfamily G (WHITE), member 2
GSH metabolism	ko00480	CL168.Contig2_All(IDH1, IDH2, icd) CL3367.Contig4_All(G6PD, zwf) Unigene17078_All(PGD, gnd, gntZ) Unigene28066_All(GST, gst) Unigene53785_All(PGD, gnd, gntZ) Unigene54130_All(RRM2) Unigene55336_All(RRM2) Unigene55337_All(RRM2)	IDH1, IDH2,icd: isocitrate dehydrogenase [EC:1.1.1.42] G6PD, zwf:glucose-6-phosphate 1-dehydrogenase [EC:1.1.1.49 1.1.1.363] PGD, gnd,gntZ:6-phosphogluconate dehydrogenase [EC:1.1.1.44 1.1.1.343] GST, gst:glutathione S-transferase [EC:2.5.1.18] RRM2: ribonucleoside-diphosphate reductase subunit M2 [EC:1.17.4.1]
Peroxisome	ko04146	CL2228.Contig2_All (SOD) CL3513.Contig1_All (CAT) Unigene16634_Al (EPHX4) Unigene1686_All (PRDX) Unigene28141_All (PRDX) Unigene2866_All (SOD) Unigene37952_All (CAT) Unigene54054_All (SOD) Unigene682_All (CAT) Unigene8435_All (SOD)	SOD: superoxide, dismutase, CAT: catalase, EPHX4: epoxide hydrolase 4, PRDX: peroxiredoxin
Phenylpropanoid biosynthesis	Ko00940	Unigene53942_All (CCR) Unigene55182_All (E1.1.1.195) Unigene54923_All (E1.1.1.195) Unigene13418_All (CCR) Unigene53862_All (E1.1.1.195) Unigene10106_All (CCR)	CCR: cinnamoyl-CoA reductase; E1.1.1.195:cinnamyl-alcohol dehydrogenase
Plant hormone signal transduction	ko04075	Unigene34841_All (ARF) CL263.Contig32_All (ARF)	ARF: auxin response factor
MAPK signaling pathway-plant	ko04016	Unigene3664_All (PAF1) Unigene55189_All (MPK4)	PAF1: RNA polymerase II-associated factor 1 MPK4: mitogen-activated protein kinase 4

## Data Availability

Data are deposited in the Mendeley Data, translated with www.DeepL.com/Translator, accessed on 1 January 2021 to 25 March 2021 (free version).
